# Efficient Fluorescence Resonance Energy Transfer between Quantum Dots and Gold Nanoparticles Based on Porous Silicon Photonic Crystal for DNA Detection

**DOI:** 10.3390/s17051078

**Published:** 2017-05-10

**Authors:** Hongyan Zhang, Jie Lv, Zhenhong Jia

**Affiliations:** 1 School of Physical Science and Technology, Xinjiang University, Urumqi 830046, China; zhanghongyanxj@163.com; 2 College of Resource and Environment science, Xinjiang University, Urumqi 830046, China; Lvjie@xju.edu.cn; 3 College of Information Science and Engineering, Xinjiang University, Urumqi 830046, China

**Keywords:** FRET, DNA biosensor, porous silicon, photonic crystal

## Abstract

A novel assembled biosensor was prepared for detecting 16S rRNA, a small-size persistent specific for Actinobacteria. The mechanism of the porous silicon (PS) photonic crystal biosensor is based on the fluorescence resonance energy transfer (FRET) between quantum dots (QDs) and gold nanoparticles (AuNPs) through DNA hybridization, where QDs act as an emission donor and AuNPs serve as a fluorescence quencher. Results showed that the photoluminescence (PL) intensity of PS photonic crystal was drastically increased when the QDs-conjugated probe DNA was adhered to the PS layer by surface modification using a standard cross-link chemistry method. The PL intensity of QDs was decreased when the addition of AuNPs-conjugated complementary 16S rRNA was dropped onto QDs-conjugated PS. Based on the analysis of different target DNA concentration, it was found that the decrease of the PL intensity showed a good linear relationship with complementary DNA concentration in a range from 0.25 to 10 μM, and the detection limit was 328.7 nM. Such an optical FRET biosensor functions on PS-based photonic crystal for DNA detection that differs from the traditional FRET, which is used only in liquid. This method will benefit the development of a new optical FRET label-free biosensor on Si substrate and has great potential in biochips based on integrated optical devices.

## 1. Introduction

Actinobacteria are widely distributed in terrestrial, freshwater and marine habitats, where they are involved in the turnover of organic matter and xenobiotic compounds [1,2]. Actinobacteria detection is highly desirable in many fields, especially in monitoring water-damaged building materials and the clinical relevance of some Actinobacteria, etc [3,4]. Molecular techniques overcome culture bias and can be used to investigate action bacteria ecology, but corresponding culture-based procedures were unsuccessful, until a new primer system (16S rRNA gene based specifically on Actinobacteria) was designed [5]. To increase the accuracy and efficiency of 16S rRNA detection, several methods have been developed. Over the last decade, label-free biosensors have been used for rapid and sensitive bimolecular detection in the monitoring of the environment, controlling of food products and their use in the medical diagnostics [6,7,8,9,10,11]. Among them, the label-free biosensor based on the hybridization between capture DNA and target DNA enables direct, sensitive and rapid detection of DNA without target amplification [12,13,14]. Various label-free sensing mechanisms are developed for DNA detection such as surface plasmon resonance (SPR), electrochemistry, reflectance spectroscopy, infrared spectroscopy and surface enhanced Raman spectroscopy (SERS) [15,16,17,18,19]. Although the above-noted methods can offer sensitive and accurate detection results, most are complicated, time-consuming or expensive. Therefore, it is of considerable significance to develop sensitive, simple and low-cost methods for DNA detection. The optical method, especially the fluorescence resonance energy transfer (FRET) method, is a significant phenomenon for probing short distance-dependent interaction of a donor–acceptor pair. This method offers operational simplicity, test rapidity, high sensitivity and the ability to measure multiple fluorescence properties [20,21]. Generally, the FRET efficiency depends on the mutual distance of the donor–acceptor pair and the spectral overlap between the emission spectrum of the donor and the absorption spectrum of the acceptor. On one hand, many organic dyes and quantum dots (QDs) have proven to be efficient FRET donors in the last several years. QDs have received considerable attention as fluorescence labels in the FRET biosensor due to its high brightness, multifunctional-group accessibility, long fluorescence lifetime and strong photoluminescence (PL) [22,23,24]. On the other hand, gold nanoparticles (AuNPs) can be considered as promising candidates for acceptors of the FRET system due to their extremely high extinction coefficient and super biocompatibility [25,26,27,28]. As a photoluminescent quencher, AuNPs can efficiently quench the molecular excitation energy in chromophore–AuNPs composite. In addition, AuNPs might be able to quench the fluorescence of QDs due to the spectral overlap of the emission of the donor and the absorption of the acceptor. Importantly, the vast potential of AuNPs in medicine and bioassays stems from the immense possibilities of designing complex architectures with precision recognition of DNA fragments. Growing interests in bioassays, providing transduction of bioinformation to optical and electronic signals have recently been observed in conjunction with stimulating developments in the synthesis of highly efficient quantum-dots and functionalized AuNPs. To the best of our knowledge, all the previously reported FRET biosensors have been focused on the traditional FRET mode in liquid [22,23,24,25,26,27]. However, those optical biosensors are limited in sensitivity and application since the surface area is small and the interaction between biomolecules and the electromagnetic field is not optimal. 

In order to increase the available surface area, enhance the field–molecule interaction strength and extend the application field of the FRET biosensor, porous materials have been proposed as an extension to biosensors. Porous silicon (PS) presents an almost ideal optical sensor platform, with potential in a diverse range of applications from trace environmental to label-free monitoring of biomolecular interaction [17,29,30]. Research interest has moved toward sensing application areas of PS because of its sponge-like structure and large internal surface-to-volume ratio which can reach several hundreds of square meters per cubic centimeter. This large value accounts for the enhanced reactivity of PS with various adsorptive substances. Furthermore, the adsorption of chemical or biological substances onto the pores of PS leads to a change in its electrical and optical properties, including its conductance, reflectance and PL properties [30,31,32,33].

Since the observation of PL from PS, there has been much interest in recent years on the structural and optical properties of PS due to its potential use in Si-based optoelectronic devices and sensors. The early stages of PS-based application research are focused mainly on the development of optoelectronic devices such as light emitting diodes (LED) and waveguides [34,35]. However, development of optoelectronic devices using a PS layer is limited by several problems. One of the main problems is that the luminescence intensity is insufficient and there is no method to control its output wavelength. In recent years, our group has invested in light-emitting devices based on PS. For example, with two different single layers forming a porous silicon Bragg reflector (DBR), a special multi-layer porous silicon photonic device was obtained and used to enhance the PL intensity of QDs, where the fluorescence peak falls into the high reflection band of Bragg reflectors [33]. Moreover, this enhancement effect can improve the performance and sensitivity of the PS biosensor.

Based on the advantages of PS biosensors, a new fluorescent PS biosensor for probing 16S rRNA was explored. Herein, we demonstrate an efficient and reliable DNA detecting system through the FRET between QDs and AuNPs based on the PS layer. It has been proved that the PL intensity of QDs is decreased when the addition of AuNPs-conjugated complementary 16S rRNA is dropped on the QDs-conjugated PS layer, which is originated from the FRET between QDs and AuNPs through DNA hybridization. For the first time, the FRET biosensor based on PS photonic crystal described in this paper shows its simplicity and flexibility. More importantly, such a highly sensitive, fast and responsive optical FRET biosensor functions in Si-based substrate for DNA detection. This biosensor will broadly benefit to develop a new optical FRET label-free biosensor on Si substrate and has great potential in biochips based on integrated optical devices.

## 2. Materials and Methods

### 2.1. Reagents and Chemicals

The oligonucleotides of 16S rRNA used in this paper were as the following sequences: probe DNA, i.e., amino-modified DNA, 5’-CAACAAGCTGATAGGCCGCC-(CH_2_)_6_-NH_2_-3’ (20 based), complementary DNA, i.e., thiol-modified DNA, 5’-CGCGGCCTATCAGCTTGTTG-(CH_2_)_6_-SH-3’ (20 based), and non-complementary DNA, i.e., thiol-modified DNA, 5’-ATTTGAACTGGTGACACGAG-(CH_2_)_6_-SH-3’ (20 based). All oligonucleotides were purchased from Invitrogen Trading Co., Ltd. (Shanghai, China).

N-(3-dimethylaminoporopyl)-N’-ethylcarbodiimide (EDC), N-hydroxysuccinimide (NHS), sodium citrate, 3-Aminopropyltriethoxysilane (APTES) and glutaraldehyde (50%, GA) were purchased from Aladdin Reagent Co. (Shanghai, China). Chloroauric acid (HAuCl_4_·4H_2_O_2_, 48%–50% Au basis) was purchased from Macklin Co., Ltd. (Shanghai, China). Phosphate buffer saline PH 7.4 (0.01 M PBS buffer solution) was obtained from Sangon Biotech Co., Ltd. (Shanghai, China).

Carboxyl-modified water-soluble quantum dots with emission at 525 nm were purchased from WuHan JiaYuan Quantum Dots Co., Ltd. (Wuhan, China). All other reagents were analytical grade and were used without further purification. Deionized water was used throughout the study.

### 2.2. Apparatus

The transmission electron microscopic (TEM) images were obtained from a JEM-2100F transmission electron microscope (Hitachi, Japan). Scanning electron micrographs (SEM) were obtained from an S-4800 scanning electron microscope (Hitachi, Japan). Fluorescence measurements were carried out on an F-4600 spectrophotometer (Hitachi, Japan). Absorption spectra were acquired on a Lambda 650 UV-vis spectrophotometer (PerkinElmer, Waltham, MA, USA) with the wavelength ranging from 200.00 to 1000.00 nm. Reflectance spectra were measured by U-4100 UV-vis scanning spectrophotometer (Hitachi, Japan) with the wavelength ranging from 400.00 to 2000.00 nm.

### 2.3. Synthesis of Citrate-Stabilized AuNPs

All glassware was immersed in freshly prepared aqua regia for 24 h, rinsed thoroughly and dried prior to use. Briefly, 20 mL of 0.5 mM HAuCl_4_ solution was heated to 90 °C under vigorous stirring for 5 min, and then 20 mL of 0.25 mM sodium citrate solution was quickly added in it. The color of the solution changed from faintly gray to claret-red. Under continuous stirring, the reaction was allowed to continue for 30 min. Then, the solution was cooled down to room temperature and stored at 4 °C in a refrigerator. 

### 2.4. AuNPs-Conjugated Target DNA

An amount of 90 μL AuNPs was mixed with different concentrations of 20 μL thiol-modified probe DNA (5’-ATTTGAACTGGTGACACGAG-(CH_2_)_6_-SH-3’) in buffer, and the mixture was incubated for 30 min with shaking and then was allowed to react for 24 h at room temperature to form AuNPs-conjugated target DNA. 

### 2.5. QD-Conjugated Probe DNA

**Figure 1:**
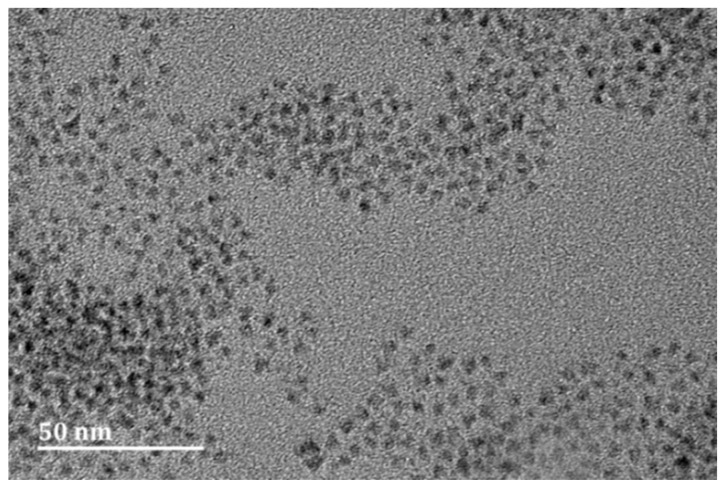
Transmission electron microscopic (TEM) image of carboxyl-modified quantum dots (QDs).

The purchased carboxyl-modified QDs with their image presented in Figure 1 have an average diameter of 5 nm. At first, the carboxyl-modified QDs solution (1 mg/mL) was sonicated for 10 min. Then, 10 mg/mL NHS solution and 10 mg/mL EDC were added into the QDs suspension (volume ratio 1:2.5:10) for 30 min. After that, amino-modified capture probe (5’-CAACAAGCTGATAGGCCGCC-(CH_2_)_6_-NH_2_-3’, 50 μM) was added into the mixture and incubated for 3 h at room temperature.

### 2.6. Preparation of Bragg Reflector

#### 2.6.1. Calculations

**Figure 2:**
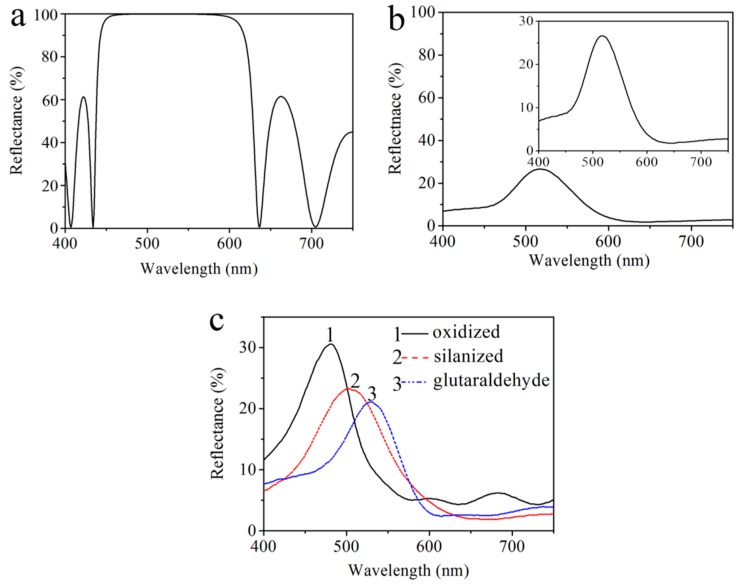
(**a**) Calculated reflectance spectrum of Bragg reflector (DBR) structure following the (n_L_n_H_)^10^ sequence; (**b**) Experimentally measured reflectance spectrum with the same sequence; (**c**) Reflectance spectra of DBRs for (1) oxidization, (2) silanization and (3) glutaraldehyde treatment.

In experiments, we chose a n-type PS DBR layer as a substrate due to its large pore size, which facilitates the penetration of biological molecules and chemical reagents. However, Macroporous silicon leads to rough and uneven areas between layers. Such a roughness in the transverse dimension of the cavity affects the spectral properties of the PS DBR, and causes a low reflectance value. Considering that too many layers can destroy the optical properties of photonic crystals, we chose the PS DBR with a sequence of (n_L_n_H_)^10^. In this study, the PS DBR was constructed with several periods of high and low porosity layers with an effective optical thickness of λ_Bragg_/4 for each DBR layer and λ_Bragg_ for the central layer, where λ_Bragg_ is the Bragg wavelength. Figure 2a shows the calculated reflectance spectra of the Bragg reflector (n_L_n_H_)^10^ by using the transfer matrix method. Following our earlier works [6,17,32], the refractive indices n_L_ and n_H_ were determined by experiments to be 1.6 and 2.6, respectively. The layer thicknesses corresponding to the refractive indices are 80 nm and 50 nm respectively (obtained from Bruggeman’s effective medium theory, where λ_Bragg_ = 520 nm). From Figure 2a, the optical characteristics of this structure become a high reflectivity stopband with a reflectance maximum at 512 nm.

#### 2.6.2. Fabrication of PS DBR

With the (n_L_n_H_)^10^ sequence, the Bragg reflector was fabricated by electrochemical etching of n-type (100) Si substrate (resistivity 0.01–0.02 Ω·cm, thickness 400 ± 10 µm). The periodic multilayer structure was fabricated by using a computer program (Labview) to alternately change the current density for different etching times with a 5-s pause after each layer formation. In experiments, the mixed solution was composed of 49% aqueous hydrofluoric, ethanol and deionized water (volume ratio 1:1:7). The effective exposed area of the Si substrate was 0.785 cm^2^ in Teflon electrochemical etch cell and the copper was immersed into the electrolyte as a counter electrode. The fabrication process began from left to right and from top to bottom into Si substrate following the sequence (n_L_n_H_)^10^, where the current density was 23 mA/cm^2^ with 3.5 s for n_L_ and was 10 mA/cm^2^ with 2.5 s for n_H_. In Figure 2b, it can be clearly seen that a reflectivity stopband at 518 nm appeared in the measured reflectance spectrum, which is in agreement to the calculated resonance peak of reflectance spectrum. However, there are some differences between the calculated and the measured reflectance spectra, especially in the low reflectance and narrow reflectivity stopband. We think these differences may come from both the uneven surface and large porosity of n-type PS, which affects the refractive index and thickness of the layer in PS DBR.

### 2.7. Functionalization of PS DBR

#### 2.7.1. Oxidization

The freshly etched PS is unstable in the air owing to the “fragile” Si–H bond, which is easily oxidized. As such, the PS surface needs to be stabilized before the immobilization. To do this, the prepared PS DBR was soaked in H_2_O_2_ (30%) for 24 h at room temperature. Then the PS substrate was rinsed with deionized water and dried in the air. In addition to stabilization, oxidation of PS also introduces hydrophilicity to the material and is an essential requirement in biological application.

#### 2.7.2. Silanization

The oxidized PS substrates were dipped into 5% solution of APTES in water/methanol mixture (v/v = 1:1) for 1 h at room temperature. In order to promote cross-linking and to remove excess solvent, the resulting substrates were then rinsed with deionized water and heated up to 100 °C for 10 min. After that, the substrates were incubated in 2.5% solution of glutaraldehyde (GA) for 1 h at room temperature. Finally, the PS substrates had to be washed three times with buffer (PH: 7.4) in order to remove all the excess of GA. 

The resulting reflectance spectra show that the high reflectivity stopband of DBR shifts from 518 nm to 482 nm after oxidation. The refractive index of silica (SiO_2_) is less than that of silicon, which results in a decrease of the refractive index of the PS layer. The high reflectivity stopband of DBR shifts from 505 nm to 531 nm, which is resulted from the increase of the refractive index of the PS layer after the silanization process and GA. The red-shifted reflectance in our experiment suggests that the small organic molecules couple well with the PS layer, and thus the functionalization has been carried out successfully. More importantly, the high reflectivity stopband at 531 nm of functionalized PS in the measured reflectance spectra is very near to the fluorescence peak of QDs, which enhances the fluorescence of QDs at 525 nm. The experimental data are given in the Figure 2c.

#### 2.7.3. Immobilization

An amount of 50 μL QD-conjugated probe DNA was dropped onto the prepared PS substrate at 37 °C and the substrate was rinsed with buffer (PH: 7.4) to remove all excess of the QD-conjugated probe DNA to prevent non-specific absorption. In this experiment, the reaction time was optimized to be 3 h.

### 2.8. Detection

The PS substrates were exposed to 50 μL solutions with the different concentration of complementary AuNPs-conjugated target DNA and incubated for 3 h at 37 °C. Finally, the PS substrates had to be washed three times with buffer in order to remove all excess of AuNPs- conjugated target DNA and dried in the air.

## 3. Results and Discussion

**Figure 3:**
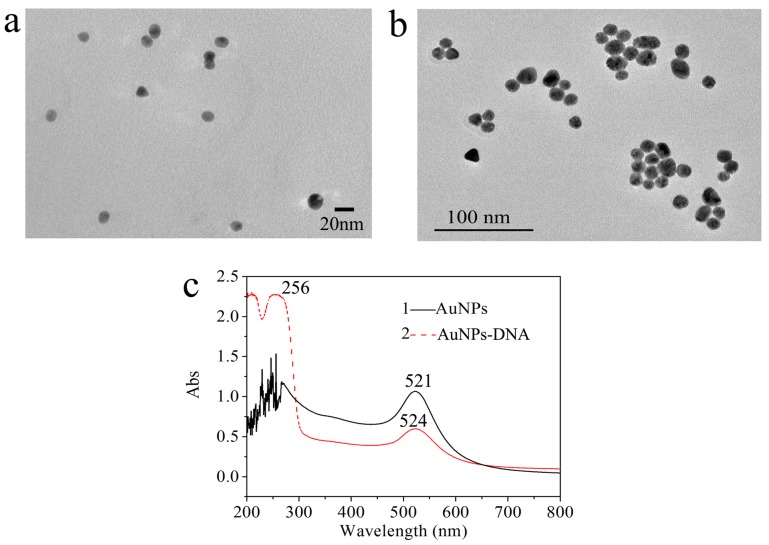
TEM images of (**a**) gold nanoparticles (AuNPs), (**b**) AuNPs-conjugated target DNA and (**c**) their relative UV-vis spectra.

The prepared AuNPs and AuNPs-conjugated target DNA were characterized by transmission electron microscopy (TEM) and UV-vis spectroscopy. The TEM experiment was performed to characterize the morphology and sizes of AuNPs. As shown in Figure 3a, the synthesized AuNPs have a good spherical shape and uniform size with an average diameter of 13 ± 2 nm. The particle size of AuNPs-conjugated target DNA was estimated to be 15 ± 2 nm from Figure 3b. In Figure 3c, the UV-visible spectrum of AuNPs exhibits a characteristic absorption peak at 521 nm. After the thiolated target DNA was dropped into colloidal AuNPs for 24 h at room temperature, the SPR absorption peak of AuNPs–DNA red-shifted slightly from 521 nm to 524 nm, which confirms the conjugation of DNA onto AuNPs and insensible enlargement of the particle size of AuNPs. Importantly, another absorption peak appearing at 256 nm is related to DNA’s characteristic absorption peak [27,36]. These effects may be due to the adsorption of DNA molecules on the surface of AuNPs via the Au–S bond.

**Figure 4:**
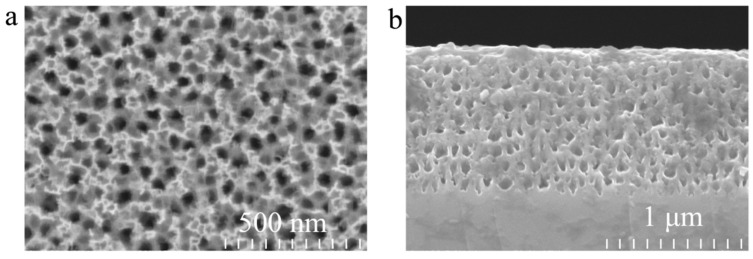
The scanning electron micrograph (SEM) image of (**a**) surface and (**b**) cross section of the porous silicon multilayer produced by electrochemical etching.

Figure 4a shows the surface image of the PS DBR layer obtained by electrochemical etching. The pore distribution is random and the average diameter of the pores is approximately 20–80 nm, which is large enough to permit easy biomolecule infiltration. Therefore, such a PS DBR layer has good capability for subsequent small molecule attachment. Figure 4b describes a cross-sectional image of the PS DBR layer. The high porosity layers correspond to the PS layers with large pore, or low refractive index (n_L_), whereas the low porosity layers correspond to the layers with small pore, or high refractive index (n_H_). The overall thickness of the PS DBR layer on the silicon wafer is approximately 1100 nm, the thickness of the low porosity layer is about 75 nm and the thickness of the high porosity layer is about 40 nm. We can see the contrast between the alternate layers of the PS DBR which confirms that a large porosity difference has been achieved. 

**Figure 5:**
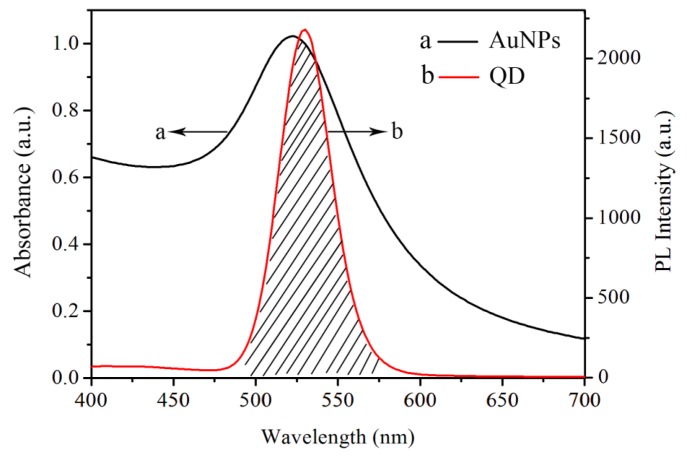
Spectra overlap between (**a**) absorption spectra of AuNPs and (**b**) emission spectra of QDs.

The efficiency of the FRET mainly depends on two factors. One is the spectral overlap between the emission of the donor and the absorption of the acceptor. The second is the distance between the donor and the acceptor. In our experiments, short distance between QD-conjugated probe DNA and AuNPs-conjugated target DNA was obtained for the FRET through 20-base pair DNA hybridization. In the FRET process, QDs transferred their fluorescence energy to the quencher AuNPs and thus their fluorescence was quenched. To further test the feasibility of energy transfer, the characteristic absorption spectrum of colloidal AuNPs and the fluorescence spectra of QDs were recorded by a UV-visible spectrophotometer (Lambda 650) as shown in Figure 5. In Figure 5a, a strong characteristic absorption peak of AuNPs appeared at 521 nm, which is in the region of the energy acceptor. Upon irradiation at 350 nm, a strong emission at 525 nm is observed in Figure 5b, which comes from the fluorescence emission from the QDs energy donor unit. Notably, the resulting absorption spectrum of the AuNPs overlaps the emission spectrum of QDs to a great extent, which means that FRET may take place between QDs as the donor and the AuNPs as the acceptor. Thus, when AuNPs-conjugated target is dropped on DBR with QD-conjugated probe DNA, AuNPs can be adsorbed onto the surface of QDs to form QDs-oligo-AuNPs assemblies due to 20-base pair DNA hybridization.

**Figure 6:**
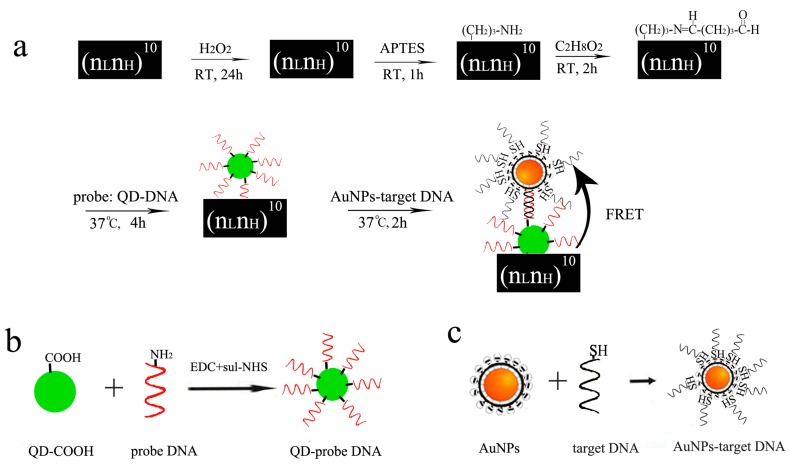
(**a**) Schematic of each functionalization step and the sensing mechanism of the proposed QDs-AuNPs fluorescence resonance energy transfer (FRET) biosensor for DNA detection; (**b**) QD-conjugated probe DNA and (**c**) AuNPs-conjugated target DNA.

Figure 6 shows the schematic of each functionalization step and the principle of the proposed FRET-based biosensor. The oxidized PS layer with Si–O–Si surface functionality reacts with APTES, then APTES attaches to the PS layer reacting further with GA, and finally GA-tagged films are covalently attached to QD-probe DNA. The chemical modification carried out through linker attachment is easy and simple. In Figure 2c, we used a UV-vis spectrophotometer to measure each functionalization step to confirm whether the chemical attachment is successful. In this proposed FRET biosensor, QD-probe DNA attached to the PS layer acts as the energy donor. When AuNPs-target DNA was added to the PS-QDs biosensor, excellent quenching efficiency through AuNPs was absorbed on the surface of QDs by DNA hybridization between probe DNA and target DNA via the FRET, which provides an ideal “off-state”. 

**Figure 7:**
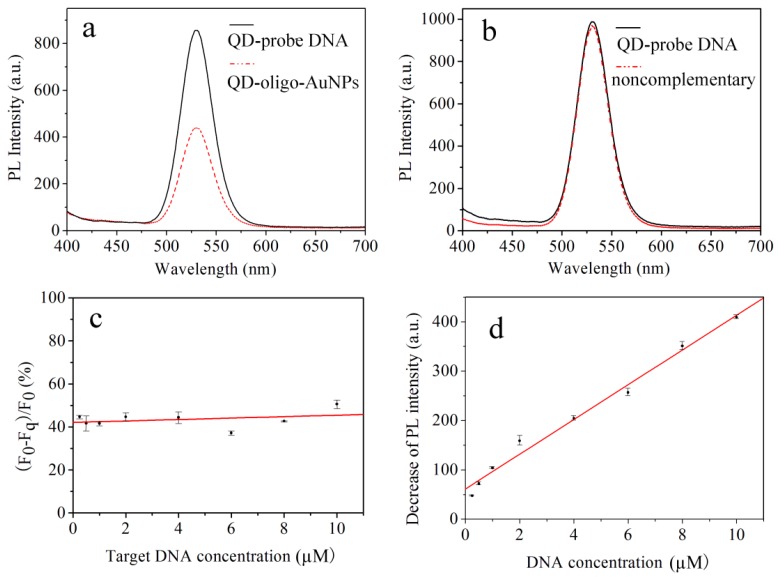
(**a**) Effects of the biosensor capped with AuNPs-conjugated complementary DNA on the fluorescence emission of QDs; (**b**) Control experiment conducted with AuNPs-conjugated noncomplementary DNA; (**c**) Quenching efficiency Q = 1 − F_q_/F_0_ with a series of target DNA concentrations; (**d**) Linear relationship between the decrease of fluorescence intensity and the AuNPs-conjugated target DNA concentration.

Our idea of FRET is based on the DNA hybridization between QDs-conjugated probe DNA and AuNPs-conjugated target DNA to form a sandwich assay structure of QDs-oligo-AuNPs. In order to further validate the method, we investigated the specificity, linear range, accuracy and detection limit. To explore the specificity of this QDs-AuNPs FRET biosensor, the control experiment was conducted with non-complementary DNA. As shown in Figure 7a, when 10 μM AuNPs-target DNA was added to the PS-QDs biosensor, the fluorescence intensity (at 532 nm) of the FRET probe was reduced from 825 (a.u.) to 445 (a.u.), which means that AuNPs can be absorbed on the surface of QDs through DNA hybridization between probe DNA and complementary DNA. All these prove that AuNPs can effectively quench QDs light emitting through 20-base pair DNA hybridization. As a result of the sandwich assay structure formation of QDs-oligo-AuNPs, QDs were close to AuNPs surface and fluorescence quenching occurred. It should be noted, from Figure 7b, that the fluorescence intensity of QDs remained unchanged with 10 μM non-complementary DNA added, since the QD-conjugated probe DNA and AuNPs-conjugated noncomplementary DNA do not bind on the surface of QDs. These results illustrate that the developed FRET probe has high specificity and selectivity for complementary DNA. To explore the quenching efficiency of this FRET biosensor, a series of concentrations from 0.25μM to 10μM of target DNA were tested. The quenching efficiency is expressed by Q = 1 − F_q_/F_0_, where F_q_ is the fluorescence intensity of QD after quenching, and F_0_ is the original fluorescence intensity of QD. As shown in Figure 7c, the quenching efficiency changes slightly when the concentration of target DNA is increased from 0.25 to 10 μM. The observed FRET efficiency increases from 35.5% to 50.6%, which is consistent with the typical quenching efficiency of the homogeneous FRET system with the range from 30% to 50%. This suggests that the concentration of the probe DNA is sufficiently able to detect different concentrations of target DNA through DNA hybridization between QD-conjugated probe DNA and AuNPs-conjugated target DNA.

Figure 7d shows the fluorescence signal quenching ∆F (∆F = F_0_ − F_q_) corresponding to different target complementary DNA concentrations, where the fluorescence signal quenching corresponding to those concentrations are 47.4 (a.u.), 72.2 (a.u.), 103.8 (a.u.), 163.2 (a.u.), 204.3 (a.u.), 251.6 (a.u.), 349.0 (a.u.), and 411.9 (a.u.), respectively. The fluorescence signal quenching is found to be a linear function of the concentration of complementary DNA in the range from 0.25 μM to 10 μM. The linear equation is ∆F = 60.77 + 35.18C (C is the concentration of complementary DNA, and ∆F is the change of fluorescence intensity at 533 nm) with the correlation coefficient R^2^ to be 0.991. The detection limit (LOD) is 328.7 nM (3σ/k, where σ is the standard deviation of blank measurements and k is the slope of the linear equation). The DNA detection limit based on the FRET biosensor that used PS as a wafer is of nM concentration, which is comparable to that reported for other favorable PS biosensor techniques based on silicon as a wafer [17]. The linear fitness of the experimental data is given in Table 1. 

## 4. Conclusions

A novel assembled FRET-based biosensor QDs-oligo-AuNPs on PS photonic crystal was designed to detect 16S rRNA, where QDs were used as a donor to label probe DNA and AuNPs were selected as an acceptor to modify target DNA. The FRET between QDs and the AuNPs results in fluorescence intensity quenching by DNA hybridization. With the optimal conditions, the fluorescence intensity of the assemble biosensor is proportional to the concentration of complement DNA in the range from 0.25 μM to 10 μM with an LOD of 328.7 nM. The FRET biosensor based on the PS photonic crystal described in this paper shows its simplicity and flexibility. More importantly, such an optical FRET biosensor is highly sensitive, fast and responsive. It also functions on PS-based photonic crystal for DNA detection, differing from the traditional FRET which is used only in liquid. This biosensor will broadly benefit the development of a new optical FRET label-free biosensor on Si substrate and has great potential in biochips based on integrated optical devices.

## Figures and Tables

**Table 1:** Sensitivity and reproducibility of the QDs-AuNP FRET biosensor to different concentrations of AuNPs-conjugated target DNA. ∆F_1_, ∆F_2_ and ∆F_3_ are fluorescence signal quenching corresponding to three samples. $\bar{\Delta F}$ is the average value of fluorescence signal quenching, s is standard deviation and RSD is relative standard deviation, where $s=\sqrt{\frac{\sum_{i=1}^{n}{\left(\Delta F_{i}-\bar{\Delta F}\right)}^{2}}{n-1}}$ $\mathrm{RSD}=\frac{s}{\bar{\Delta F}}\times 100\%$.

Target DNA	∆F (a.u.)			$\bar{\Delta F}$ (a.u.)	s (a.u.)	RSD	$\bar{\Delta F}$ ± s (a.u.)
	∆F_1_	∆F_2_	∆F_3_				
Complementary DNA (0.25 μM)	45.8	47.9	49.3	47.4	1.8	3.8%	47.4 ± 1.8
Complementary DNA (0.5 μM)	70.0	77.3	69.4	72.2	4.4	6.0%	72.2 ± 4.4
Complementary DNA (1.0 μM)	103.0	101.9	106.6	103.8	2.5	2.4%	103.8 ± 2.5
Complementary DNA (2.0 μM)	148.9	168.8	171.9	163.2	12.5	7.7%	163.2 ± 12.5
Complementary DNA (4.0 μM)	201.1	211.0	200.9	204.3	5.8	2.8%	204.3 ± 5.8
Complementary DNA (6.0 μM)	253.2	246.7	255.0	251.6	4.4	1.7%	251.6 ± 4.4
Complementary DNA (8.0 μM)	347.8	353.1	346.1	349.0	3.7	1.1%	349.0 ± 3.7
Complementary DNA (10.0 μM)	409.9	410.5	415.3	411.9	3.0	0.7%	411.9 ± 3.0

## References

[1] Stach J.E.M., Maldonado L.A., Ward A.C., Goodfellow M., Bull A.T. (2003). New primers for the class Actinobacteria: Application to marine and terrestrial environments. Environ. Microbiol..

[2] Qin S., Miao Q., Feng W.W., Wang Y., Zhu X., Xing K., Jiang J.H. (2015). Biodiversity and plant growth promoting traits of culturable endophytic Actinobacteria associated with *Jatropha curcas* L. growing in Panxi dry-hot valley soil. Appl. Soil. Ecol..

[3] Ettoumi B., Chouchane H., Guesmi A., Mahjoubi M., Brusetti L., Neifar M., Borin S., Daffonchio D., Cherif A. (2016). Diversity, ecological distribution and biotechnological potential of Actinobacteria inhabiting seamounts and non-seamounts in the Tyrrhenian Sea. Microbiol. Res..

[4] Bilyk O., Luzhetskyy A. (2016). Metabolic engineering of natural product biosynthesis in Actinobacteria. Curr. Opin. Biotechnol..

[5] Schäfer J., Jäckel U., Kämpfer P. (2010). Development of a new PCR primer system for selective amplification of Actinobacteria. FEMS. Microbiol. Lett..

[6] Zhang H.Y., Jia Z.H., Lv X.Y. (2015). Surface layer reflective index changes of Au nanoparticle functionalized porous silicon microcavity for DNA detection. Curr. Appl. Phys..

[7] Shi Y., Pan Y., Zhang H., Zhang Z., Li M.J., Yi C., Yang M. (2014). A dual-mode nanosensor based on carbon quantum dots and gold nanoparticles for discriminative detection of glutathione in human plasma. Biosens. Bioelectron..

[8] Yin Z., Li H., Xu W., Cui S., Zhou D., Chen X., Zhu Y., Qin G., Song H. (2016). Local field modulation induced three-order upconversion enhancement: Combining surface plasmon effect and photonic crystal effect. Adv. Mater..

[9] Salamon H., Skopić M.K., Jung K., Bugain O., Brunschweiger A. (2016). Chemical biology probes from advanced DNA-encoded libraries. ACS Chem. Biol..

[10] Xu S., Xu W., Wang Y.F., Zhang S., Zhu Y.S., Tao L., Xia L., Zhou P.W., Song H.W. (2014). NaYF_4_:Yb,Tm nanocrystals and TiO_2_ inverse opal composite films: A novel device for upconversion enhancement and solid-based sensing of avidin. Nanoscale.

[11] Zhu Y., Cui S., Wang Y., Liu M., Lu C., Mishra A., Xu W. (2016). Enhanced rare earth photoluminescence in inverse opal photonic crystals and its application for pH sensing. Nanotechnology.

[12] Mariani S., Scarano S., Spadavecchia J., Minunni M. (2015). A reusable optical biosensor for the ultrasensitive and selective detection of unamplified human genomic DNA with gold nanostars. Biosens. Bioelectron..

[13] Zhou R., Xu C., Dong J., Wang G. (2015). Labeling-free fluorescent detection of DNA hybridization through FRET from pyrene excimer to DNA intercalator SYBR green I. Biosens. Bioelectron..

[14] Zhang Y., Geng X., Ai J., Gao Q., Qi H., Zhang C. (2015). Signal amplification detection of DNA using a sensor fabricated by one-step covalent immobilization of amino-terminated probe DNA onto the polydopamine-modified screen-printed carbon. Sens. Actuators B Chem..

[15] Cheng X.R., Hau B.Y.H., Endo T., Kerman K. (2014). Au nanoparticle-modified DNA sensor based on simultaneous electrochemical impedance spectroscopy and localized surface plasmon resonance. Biosens. Bioelectron..

[16] Li Z., Miao X., Xing K., Zhu A., Ling L. (2015). Enhanced electrochemical recognition of double-stranded DNA by using hybridization chain reaction and positively charged gold nanoparticles. Biosens. Bioelectron..

[17] Zhang H., Jia Z., Lv X., Zhou J., Chen L., Liu R., Ma J. (2013). Porous silicon optical microcavity biosensor on silicon-on-insulator wafer for sensitive DNA detection. Biosens. Bioelectron..

[18] Ilkhani H., Hughes T., Li J., Zhong C.J., Hepel M. (2016). Nanostructured SERS-electrochemical biosensors for testing of anticancer drug interactions with DNA. Biosens. Bioelectron..

[19] Kurbanoglu S., Dogan-Topal B., Rodriguez E.P., Palabiyik B.B., Ozkan S.A., Uslu B. (2016). Advances in electrochemical DNA biosensors and their interaction mechanism with pharmaceuticals. J. Electroanal. Chem..

[20] Li T., Byun J.Y., Kim B.B., Shin Y.B., Kim M.G. (2013). Label-free homogeneous FRET immunoassay for the detection of mycotoxins that utilizes quenching of the intrinsic fluorescence of antibodies. Biosens. Bioelectron..

[21] Huang X., Huang X., Zhang A., Zhuo B., Lu F., Chen Y., Gao W. (2015). Quenching of the electrochemiluminescence of RU-complex tagged shared-stem hairpin probes by graphene oxide and its application to quantitative turn-on detection of DNA. Biosens. Bioelectron..

[22] Saha J., Roy A.D., Dey D., Chakraborty S., Bhattacharjee D., Paul P.K., Hussain S.A. (2015). Investigation of Fluorescence Resonance Energy Transfer between Fluoresce in and Rhodamine 6G. Spectrochim. Acta A.

[23] Esteve-Turrillas F.A., Abad-Fuentes A. (2013). Applications of quantum dots as probes in immunosensing of small-sized analytes. Biosens. Bioelectron..

[24] Dai H., Shi Y., Wang Y., Sun Y., Hu J., Ni P., Li Z. (2014). A carbon dot based biosensor for melamine detection by fluorescence resonance energy transfer. Sens. Actuators B Chem..

[25] Li S., Qiu W., Zhang X., Ni J., Gao F., Wang Q. (2016). A high-performance DNA biosensor based on the assembly of gold nanoparticles on the terminal of hairpin-structured probe DNA. Sens. Actuators B Chem..

[26] Xu X., Qiao J., Li N., Qi L., Zhang S. (2015). Fluorescent probe for turn-on sensing of L-cysteine by ensemble of AuNCs and polymer protected AuNPs. Anal. Chim. Acta.

[27] Bu D., Zhuang H., Yang G., Ping X. (2014). An immunosensor designed for polybrominated biphenyl detection based on fluorescence resonance energy transfer (FRET) between carbon dots and gold nanoparticles. Sens. Actuators B Chem..

[28] Mohammed A.M. (2016). Fabrication and characterization of gold nano particles for DNA biosensor applications. Chin. Chem. Lett..

[29] Haidary S.M., Mohammed A.B., Córcoles E.P., Ali N.K., Ahmad M.R. (2016). Effect of coatings and surface modification on porous silicon nanoparticles for delivery of the anticancer drug tamoxifen. Microelectron. Eng..

[30] Hiraoui M., Haji L., Guendouz M., Lorrain N., Moadhen A., Oueslati M. (2012). Towards a biosensor based on anti resonant reflecting optical waveguide fabricated from porous silicon. Biosens. Bioelectron..

[31] Wei X., Weiss S.M. (2011). Guided mode biosensor based on grating coupled porous silicon wavelength. Opt. Express.

[32] Sanger A., Kumar A., Chauhan S., Gautam Y.K., Chandra R. (2015). Fast and reversible hydrogen sensing properties of Pd/Mg thin film modified by hydrophobic porous silicon substrate. Sens. Actuators B Chem..

[33] Liu C., Jia Z., Lv X., Lv C., Shi F. (2015). Enhancement of QDs’ fluorescence based on porous silicon Bragg reflector. Phys. B.

[34] Kim H.G., Lee K.W. (2015). Electrostatic gas sensor with a porous silicon diaphragm. Sens. Actuators B Chem..

[35] Park J., Yanagida Y., Hatsuzawa T. (2016). Fabrication of p-type porous silicon using double tank electrochemical cell with halogen and LED light sources. Sens. Actuators B Chem..

[36] Miao Y., Gan N., Li T., Cao Y., Hu F., Chen Y. (2016). An ultrasensitive fluorescence aptasensor for chloramphenicol based on FRET between quantum dots as donor and the magnetic SiO_2_@AuNPs probe as acceptor with exonuclease-assisted target recycling. Sens. Actuators B Chem..

